# Resolvin D1 suppresses inflammation in human fibroblast-like synoviocytes via the p-38, NF-κB, and AKT signaling pathways

**DOI:** 10.1007/s11626-024-01008-9

**Published:** 2025-03-10

**Authors:** Makoto Yanoshita, Naoto Hirose, Sayuri Nishiyama, Eri Tsuboi, Naoki Kubo, Daiki Kita, Kotaro Tanimoto

**Affiliations:** Department of Orthodontics and Craniofacial Developmental Biology, Hiroshima University Graduate School of Biomedical and Health Sciences, Kasumi 1-2-3 Minami-Ku, Hiroshima-Shi, Hiroshima Prefecture, Japan

**Keywords:** Fibroblast-like synoviocytes, Osteoarthritis, Resolvin D1, Inflammatory disorders, Endogenous docosahexaenoic acid-derived lipid mediator

## Abstract

Synovitis represents the initial pathological change in osteoarthritis and contributes to its progression. Resolvin D1 (RV-D1) is a novel and endogenous docosahexaenoic acid-derived lipid mediator, which regulates the duration and magnitude of inflammation by downregulating pro-inflammatory genes and mediators. However, the effects of RV-D1 on synovitis remain unknown. The aim of the present study was to investigate the anti-inflammatory effects of RV-D1 in human fibroblast-like synoviocytes (HFLSs) and the underlying mechanisms. The expression of the HFLS formyl peptide receptor 2 (ALX/FPR) was examined via immunocytochemical analysis. HFLSs were treated with 1 ng/mL recombinant human interleukin-1β (IL-1β) and RV-D1. The gene expression of interleukin-1β (*IL1B*), matrix metalloproteinase 3 (*MMP3*), and *MMP13* was examined using real-time reverse transcription-polymerase chain reaction after treatment with IL-1β and RV-D1. The effect of RV-D1 on apoptosis was examined based on fluorescence intensity. Phosphorylation of p-38, extracellular signal-regulated kinase, c-Jun N-terminal kinase, nuclear factor kappa B (NF-κB), and AKT was analyzed via western blotting. ALX/FPR staining was observed on the cell surface. RV-D1 significantly suppressed the IL-1β-induced increase in gene and protein expression of IL-1β, MMP-3, and MMP-13. Pretreatment with 100 nM RV-D1 significantly increased the fluorescence intensity compared to that in the non-treatment group. Furthermore, pretreatment with RV-D1 significantly suppressed the phosphorylation of p-38, NF-κB, and AKT. Whereas WRW4, an antagonist of ALX/ FPR2, treatment weakened the effect of RV-D1, resulting in p-38, NF-κB, and AKT phosphorylation and the protein expression of MMP-13 at levels comparable to those in the IL-1β without RV-D1. In conclusion, RV-D1 suppressed IL-1β and MMP expression by inhibiting the phosphorylation of p-38, NF-κB, and AKT in inflammation in HFLSs. RV-D1 can be used to develop treatments for osteoarthritis and other inflammatory disorders.

## Introduction

Osteoarthritis (OA) is a degenerative joint disease characterized by cartilage loss, subchondral bone remodeling, and synovitis (Glyn-Jones *et al*. 2015). OA is generally managed via oral nonsteroidal anti-inflammatory drugs (NSAIDs) and hyaluronic acid injections (van der Heide *et al*. 1996). However, the use of NSAIDs is associated with potentially significant gastrointestinal toxicity (Hijos-Mallada *et al*. 2022), and many individuals experience pain, stiffness, or swelling in their joints after hyaluronic acid injections (Aggarwal and Sempowski 2004).

Synovitis represents the initial pathological change in OA and contributes to its progression. Fibroblast-like synoviocytes (FLSs) release inflammatory mediators and cartilage-degrading factors such as interleukin-1 beta (IL‑1β) and matrix metalloproteinases (MMPs), which subsequently enhance inflammation, forming a vicious cycle (Atukorala *et al*. 2016). Therefore, it is hypothesized that the suppression of synovial inflammation will prevent the vicious cycle that starts with inflammation and progressing to cartilage destruction. In recent years, it has been reported that the transition from inflammatory to non-inflammatory states is not passive, but rather an active regulatory program designated as the resolution of inflammation (Serhan 2007). Resolution of inflammation is widely accepted to be a programmed, self-limiting process controlled by a group of bioactive lipid mediators known as specialized pro-resolving lipid mediators (SPMs). SPMs are produced from omega-3 essential fatty acids, such as docosahexaenoic acid (DHA) and eicosapentaenoic acid. Resolvin D (RV-D) is the final biosynthetic product of the omega-3 polyunsaturated fatty acid, DHA, produced by lipoxygenase-mediated metabolism. Rv-D binds to the G protein-coupled receptor 32 (GPR32) or formyl peptide receptor 2 (ALX/FPR2) receptors and promotes degradation (Alqahtani *et al*. 2022). Rv-D functions as a potent agonist that modulates the duration and intensity of inflammation by decreasing the expression of pro-inflammatory genes and mediators. In addition, it can suppress the recruitment of neutrophils motility to inflammation sites, accelerate the removal of apoptotic cells and microbes, and decrease exudate production. Thus, Rv-D prevents excessive inflammation by accelerating the recovery of tissue homeostasis (Serhan *et al*. 2011). Notably, RV-D1 is highly expressed in the knee synovial fluid of knee OA patients (Benabdoune *et al*. 2016). Moreover, RV-D1 alleviates bone and joint destruction in arthritic mice, reduces the levels of inflammatory mediators, and markedly suppresses the turnover of bone and cartilage, suggesting its potential in the treatment of inflammatory arthritis and related osteochondral disorders (Benabdoun *et al*. 2019). However, the effects of RV-D1 on synovitis, an early response to OA, and the underlying mechanisms remain to be elucidated. Therefore, the anti-inflammatory effects of RV-D1 on human FLSs (HFLSs) and its mechanisms were investigated.

## Materials and methods

### Cell culture

HFLSs were obtained from Cell Applications (San Diego, CA) in February 2024 and seeded in 100-mm dishes (Corning, Corning, NY) at a density of 1 × 10^4^ cells/cm^2^. The cells were incubated in a humidified environment at 37 °C with 5% CO_2_ in 10 mL α-modified Eagle’s medium (Sigma Aldrich, St. Louis, MO) containing 10% fetal bovine serum (Sigma Aldrich), 50 µg/mL penicillin (Meiji Seika, Tokyo, Japan), and 60 µg/mL kanamycin (Meiji Seika).

The expression of signaling proteins was maximally elevated 10 min following IL-1β (Fujifilm, Osaka, Japan) treatment for HFLS, while RNA expression of MMP-3 and MMP-13 reached its peak at 12 h, and their protein levels were most significantly increased at 24 h. Therefore, the cells were treated with 1 ng/mL recombinant human IL-1β for 12 h for molecular analysis. The cells were treated with IL-1β for 10 min for expression analysis of signaling pathway proteins, and the cells were treated for 24 h for IL-1β and MMP protein analysis.

The cells were then treated with RV-D1 (Cat#10,012,554, Cayman, MI) and or 10 μM WRW4 (Cat#S9818, Selleck, TX, USA), an antagonist of ALX/ FPR2, for 2 h prior to treatment with IL-1β.

All cells used in the experiment were mycoplasma-free cells, and mycoplasma contamination was examined by PCR every month. Cells were last tested at Hiroshima University in July 2024.

### Immunocytochemical staining

HFLSs were fixed with 4% paraformaldehyde in 0.1% Triton/PBS for 15 min and blocked with 1% bovine serum albumin (BSA)/PBS in phosphate-buffered saline for 60 min. Next, a 1:100 dilution of anti-ALX/FPR2 (Cat# ab203129; Abcam, Cambridge, United Kingdom) and anti-GPR32 (Cat# ab79516, Abcam) antibodies in 1% BSA/PBS were added and incubated at 4 °C overnight. After washing with PBS, we used Alexa Fluor 594 Goat anti- Rat IgG Secondary antibodies (Cat # A-11007; Thermo Fisher Scientific, Waltham, MA) with 4′,6-diamidino-2-phenylindole (DAPI) for 1 h at room temperature.

### Fluorescence intensities of SYTOX green-stained apoptotic cells

HFLSs were seeded in 24-well cell culture dishes and cultured until they reached 80% confluency, after which RV-D1 was added at concentrations of 0, 0.1, 1, 10, and 100 nM. Subsequently, SYTOX green (Cat#S7020, Thermo Fisher Scientific) was added and incubated for 24 h in a CELLCYTE X Live Cell Analyzer (Echo, San Diego, CA) equipped with a fluorescence detection device. Apoptosis was assessed by measuring the area fluorescing at 523 nm in an area of 3.72 mm^2^ at two random locations from each well.

### Real-time RT-PCR

Total RNA was extracted from HFLSs using a TRIzol RNA Isolation Reagents (Invitrogen, Carlsbad, MA). Total RNA concentration was measured using a NanoDrop (NanoDrop, ThermoScientific) and cDNA was synthesized from 1 µg of total RNA using ReverTra Ace® (Toyobo, Osaka, Japan) and random primers (Toyobo). Then, according to the specific primers (Table 1), the expression levels of each gene were analyzed by real-time RT-PCR using the LightCycler®480 real-time PCR system (Roche Diagnostic, Basel, Switzerland). Relative gene expression levels in each sample were calculated as the ratio of target gene expression to that of β-actin. All experiments were carried out in triplicate to ensure accuracy, and the results were averaged.

**Table 1.** Primer sequences for real-time reverse transcription-polymerase chain reaction

Gene	Sequence 5’ - 3’	
IL-1β	Forward	GGGGTACCGGGAGCCAAATTAAAAT
	Reverse	CCGCTCGAGCCAGATGAGCTCTGCC
MMP-3	Forward	AGTCTTCCAATCCTACTGTTGCT
	Reverse	TCCCCGTCACCTCCAATCC
MMP-13	Forward	CCCCAGGCATCACCATTCAA
	Reverse	CAGGTAGCGCTCTGCAAACT
β-actin	Forward	GTCGTACCACTGGCATTGTG
	Reverse	TCTCAGCTGTGGTGGTGAAG

### Western blot analysis

Proteins were extracted from HFLSs using Triton buffer (50 mM Tris, 250 mM NaCl, 0.1% Triton X-100, 1 mM EDTA, and 50 mM NaF) with the protease inhibitor (Sigma Aldrich, St. Louis, MO). Twenty micrograms of protein was loaded into each well and further separated based on molecular weight by sodium dodecyl sulfate–polyacrylamide gel electrophoresis (ATTO, Tokyo, Japan). After electrophoresis, the proteins were electro-transferred for 60 min to PVDF membranes. The membranes were blocked with 3% nonfat dry milk (Cell Signaling Technology, Danvers, MA) and incubated with primary antibodies: anti-IL-1β (Cat# 9211, Cell Signaling Technology), anti-MMP-3 (Cat# 8690, Cell Signaling Technology), anti-MMP-13 (Cat# 4370, Cell Signaling Technology), anti-phosphorylated p-38 (Cat# 9211, Cell Signaling Technology), anti-total p-38 (Cat# 8690, Cell Signaling Technology), anti-phosphorylated extracellular signal-regulated kinase (ERK1/2; Cat# 4370, Cell Signaling Technology), anti-total ERK1/2 (Cat# 4695, Cell Signaling Technology), anti-phosphorylated c-Jun N-terminal kinase (JNK; Cat# 4668S, Cell Signaling Technology), anti-total JNK (Cat# 9252, Cell Signaling Technology), anti-phosphorylated AKT (Cat# 4051S, Cell Signaling Technology), anti-total Akt (Cat# 9272S, Cell Signaling Technology), anti-phosphorylated nuclear factor kappa B (NF-κB) p65 (Cat# 3033S, Cell Signaling Technology), anti-total NF-κB p65 (Cat# 8242, Cell Signaling Technology), and anti-β-actin (Cat# 4970, Cell Signaling Technology). The protein bands were scanned using Image Studio software (LI-COR, Lincoln, NE) and normalized by β-actin as a control.

### Statistical analysis

All experiments were conducted in triplicate. All data were presented as the mean ± standard deviation, and values of *p* < 0.05 were considered significant. For multiple comparisons, significance was determined using ANOVA followed by Tukey–Kramer multiple comparisons post-hoc analysis (Statcel version 3 (OMS publishing, Saitama, Japan).

## Results

### Expression of the RV-D1 receptor ALX/FPR2 in HFLSs

**Figure 1.**
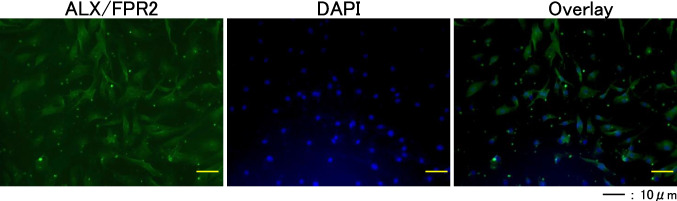
Immunocytochemistry for ALX/FPR2 in human fibroblast-like synoviocytes. Immunofluorescence staining for ALX/FPR2. Nuclei were stained with DAPI. ALX/FPR staining was observed on the cell surface. *Scale bar* represents 100 µm in each *panel*.

Immunocytochemical staining was performed to assess the expression of ALX/FPR2 and GPR32 proteins in cultured HFLSs. ALX/FPR2 staining was observed on the cell surface (Fig. 1), whereas GPR32 staining was not observed (data not shown).

### Effect of RV-D1 on IL-1β-induced MMPs expression

**Figure 2.**
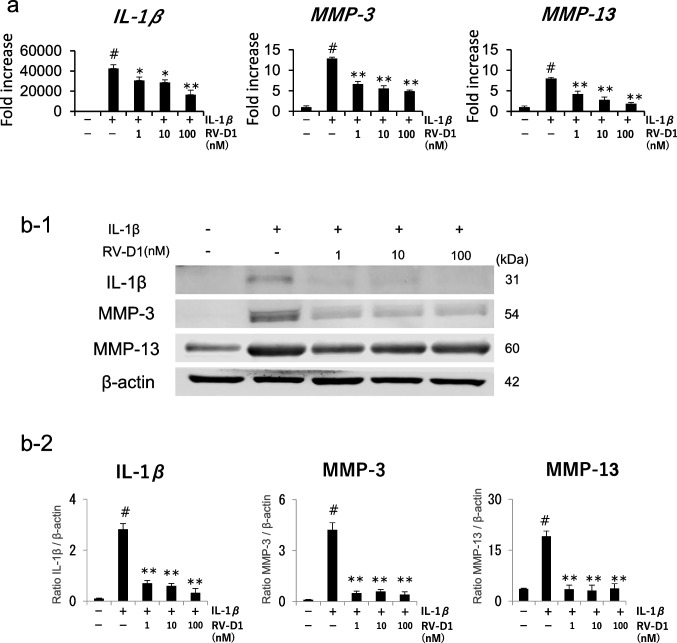
Effect of RV-D1 on IL-1β-treated MMPs. **(a)** Human fibroblast-like synoviocytes were pretreated with RV-D1 (1, 10, or 100 μM) for 2 h followed by IL-1β administration, and gene expression of *IL1B*, *MMP3*, and *MMP13* was measured at each time point using real-time reverse transcription-polymerase chain reaction. Relative mRNA levels were calculated as the ratio to that of β-actin. **(b-1)** Proteins were extracted 24 h after IL-1β was added. Similar to gene expression, RV-D1 decreased the protein expression of IL-1β, MMP-3, and MMP-13. **(b-2)** Relative protein expression was quantified using Image Studio software. Pretreatment with RV-D1 significantly suppressed the protein expression of IL-1β, MMP-3, and MMP-13. Data are presented as the mean ± standard deviation of the three independent experiments and differences between mean values were assessed by ANOVA using Tukey–Kramer multiple comparisons post-hoc analysis (# *p* < 0.01 vs. IL-1β (-), RV-D1 (-), * *p* < .0.05, **p < 0.01 vs. IL-1β ( +), RV-D1 (-)).

To validate the anti-inflammatory properties of RV-D1, the cells were pre-treated with RV-D1 2 h before treatment with IL-1β. The gene expression of *IL1B, MMP3,* and *MMP13* was assessed 12 h post-treatment, while protein expression was evaluated 24 h later. RV-D1 treatment significantly suppressed the IL-1β-induced upregulation of *IL1B*, *MMP3*, and *MMP13* (Fig. 2*a*). Furthermore, RV-D1 significantly suppressed the protein expression levels (Fig. 2*b*).

### Effect of RV-D1 on cytotoxicity

**Figure 3.**
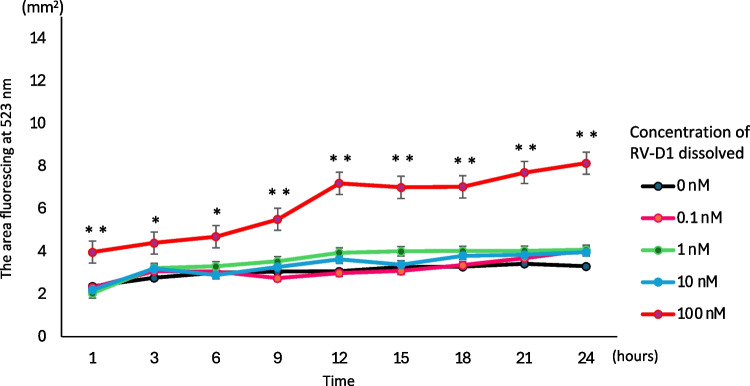
Effect of RV-D1 on cytotoxicity. Human fibroblast-like synoviocytes were exposed to RV-D1 with SYTOX and incubated for 24 h. The group exposed to 100 nM RV-D1 showed greater cell death at all time points compared to that of the control group. The data are presented as the mean ± standard deviation of three independent experiments, and differences between mean values were assessed using Tukey–Kramer multiple comparisons post-hoc analysis (*p < 0.05, **p < 0.01).

To assess the effect of RV-D1 treatment on apoptosis, the cells were treated with SYTOX 1 h after treatment with RV-D1 and then incubated for 24 h. The fluorescent regions were subsequently juxtaposed for comparison. Throughout all intervals, pretreatment with 100 nM RV-D1 significantly increased the fluorescence intensity compared to that observed after treatment with 0 nM. Except for 3 h after the administration of IL-1β, pretreatment with 100 nM RV-D1 significantly increased the fluorescence intensity compared to that in the 0.1, 1, and 10 nM groups (Fig. 3). In subsequent experiments, RV-D1 was administered at a concentration of 10 nM.

### Effects of RV-D1 on IL-1β-activated mitogen-activated protein kinase, NF-κB, and AKT signaling pathways

**Figure 4.**
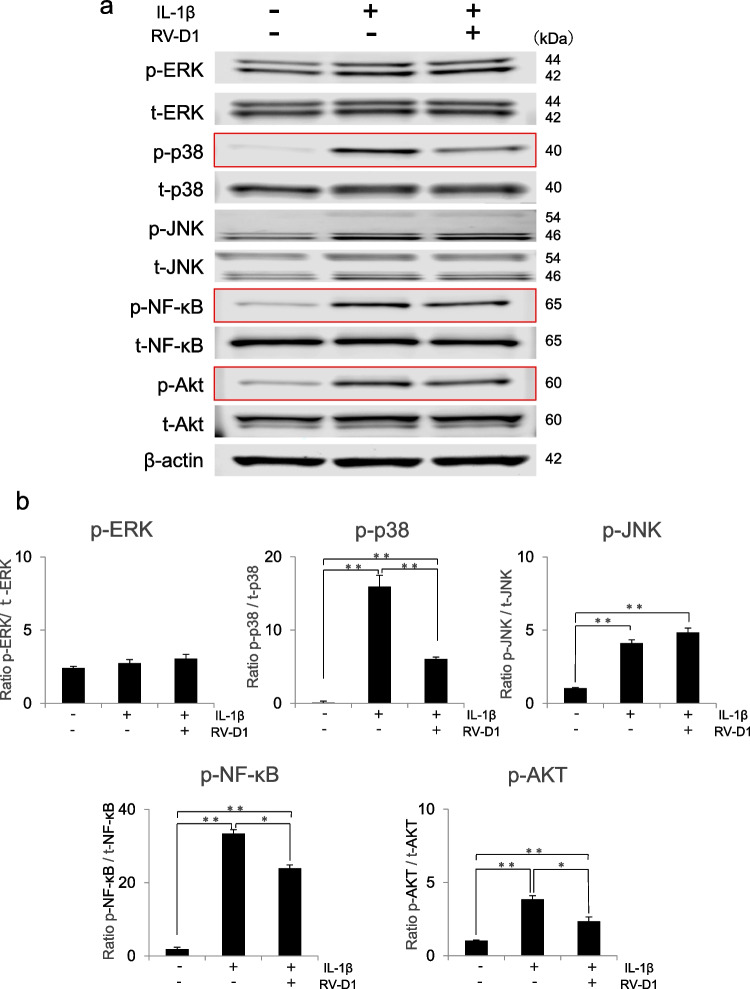
Effects of RV-D1 on IL-1β-activated signaling pathways. **(a)** Human fibroblast-like synoviocytes were pretreated with RV-D1 (10 nM) for 2 h followed by IL-1β administration, after which p-38, ERK, JNK, NF-κB, and AKT phosphorylation was examined by western blot analysis after 10 min of exposure to IL-1β. (**b)** Relative protein expression was quantified using Image Studio software. The data are presented as the mean ± standard deviation of three independent experiments and differences between mean values were assessed using Tukey–Kramer multiple comparisons post-hoc analysis (*p < 0.05, **p < 0.01). Pretreatment with RV-D1 significantly decreased the phosphorylation of p-38, NF-κB, and AKT.

To investigate the effect of RV-D1 on mitogen-activated protein kinases (MAPKs), NF-κB, and AKT signaling, HFLSs were treated with IL-1β for 10 min following a 2-h pre-treatment with RV-D1. Pretreatment with RV-D1 significantly decreased the phosphorylation of p-38, NF-κB, and AKT (Fig. 4). IL-1β treatment significantly activated the phosphorylation of JNK. However, RV-D1 treatment did not affect JNK phosphorylation.

**Figure 5.**
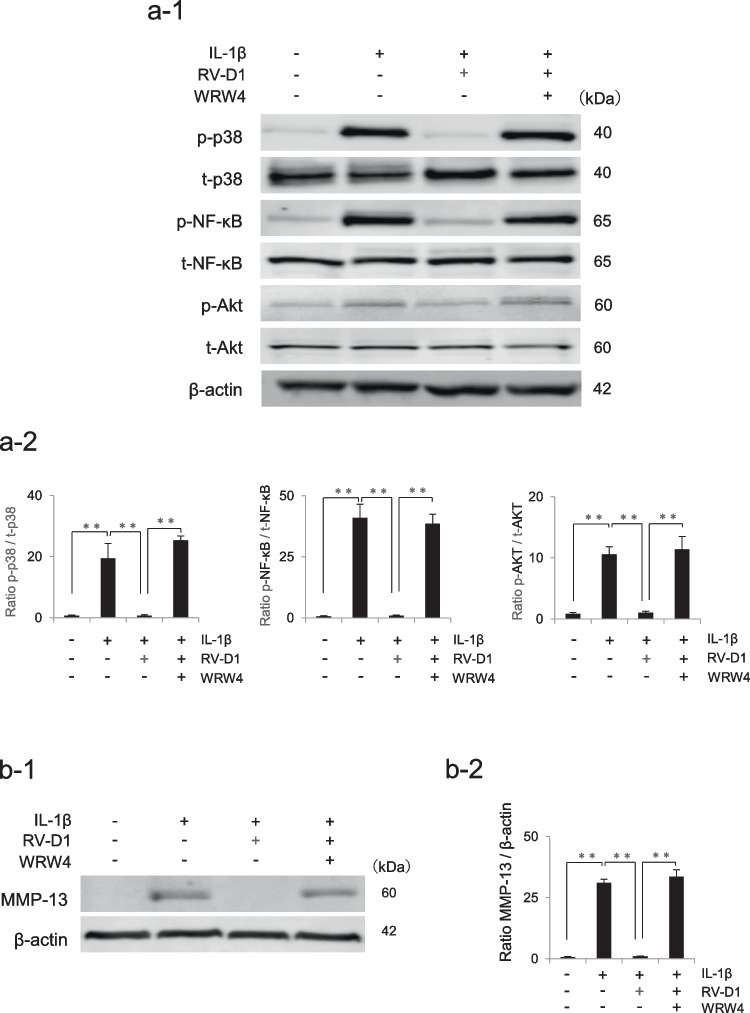
Effect of WRW4, ALX/FPR2 antagonist, on the phosphorylation of p-38, NF-κB, and AKT and MMP-13 expression suppressed by RV-D1. **(*a-1*)** Human fibroblast-like synoviocytes were pretreated with RV-D1 (10 nM) and or WRW4 (10 μM) for 2 h followed by IL-1β administration for 10 min, after which p-38, NF-κB, and AKT phosphorylation was examined by western blot analysis. **(*a-2*)** Pretreatment with RV-D1 and WRW4 stimulated p-38, NF-κB, and AKT phosphorylation suppressed by RV-D1.** (*b-1*)** Human fibroblast-like synoviocytes were pretreated with RV-D1 (10 nM) and or WRW4 (10 μM) for 2 h followed by IL-1β administration for 24 h, after which the protein expression of MMP-13 was examined by western blot analysis. **(*b-2*)** The suppression of MMP13 expression by RV-D1 was significantly attenuated by WRW4 treatment. Relative protein expression was quantified using Image Studio software. The data are presented as the mean ± standard deviation of three independent experiments and differences between mean values were assessed using Tukey–Kramer multiple comparisons post-hoc analysis (**p < 0.01).

To prove that RV-D1 binds to ALX/FPR2 and suppresses MMP-13 expression by regulating p-38, NF-κB, and AKT signaling pathways, the protein expressions of p-38, NF-κB, AKT and MMP13 were compared among four groups: control, with IL-1β, with RV-D1, and with RV-D1 and WRW4, an antagonist of ALX/ FPR2. Pretreatment with RV-D1 and WRW4 stimulated the phosphorylation of p-38, NF-κB, and AKT significantly suppressed by RV-D1 after 10 min exposure to IL-1β (Fig. 5*a*). Moreover, WRW4 treatment weakened the effect of RV-D1, resulting in the protein expression of MMP-13 at levels comparable to those in the IL-1β without RV-D1 (Fig. 5*b*).

**Figure 6.**
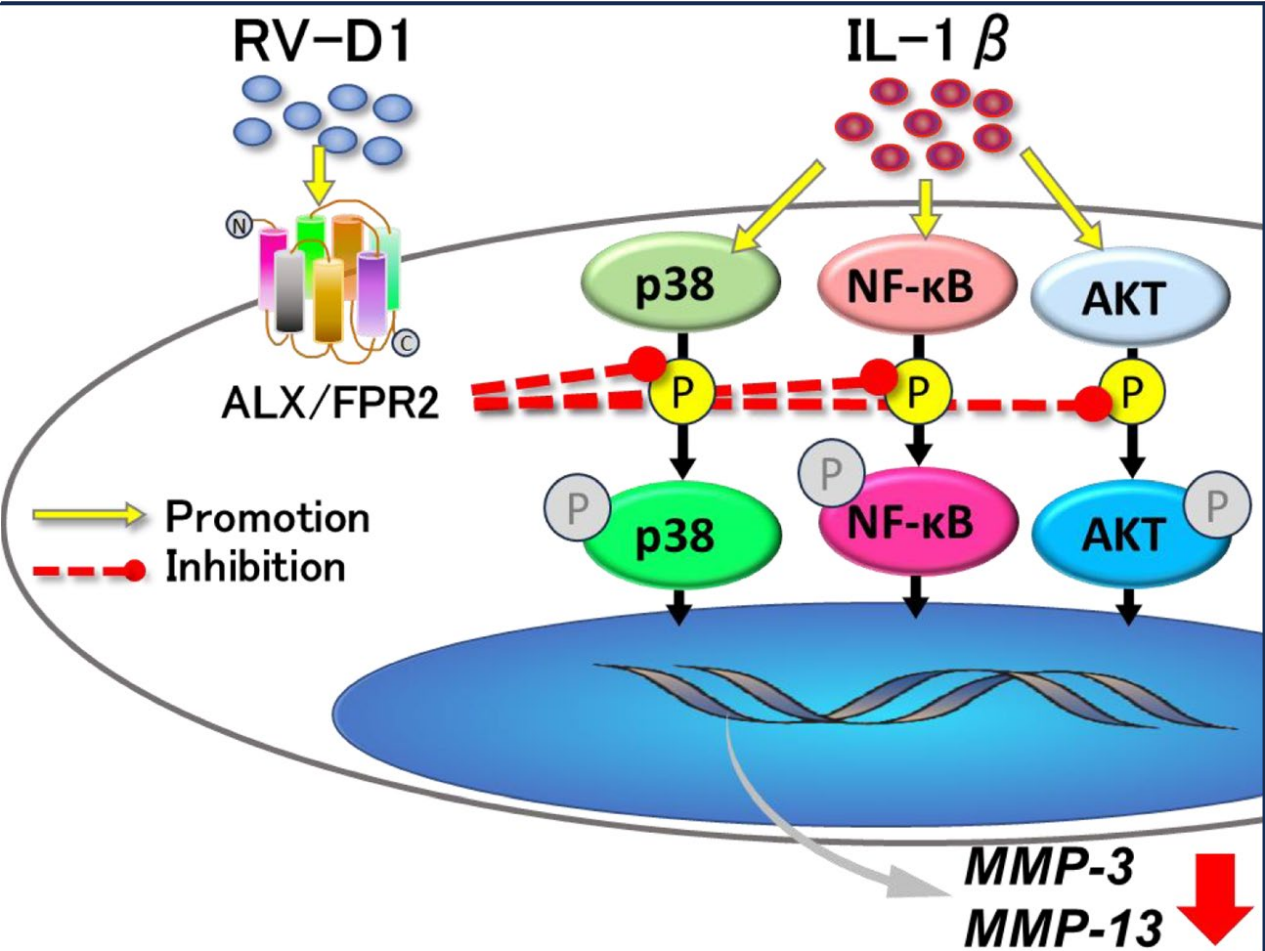
Graphical summarization of the functions of the Rv-D1-ALX/FPR2 interaction on Human fibroblast-like synoviocytes. The RV-D1-ALX/FPR2 interaction can suppresses inflammation and decrease MMP-3 and MMP-13 by regulating p-38, NF-κB, and AKT phosphorylation.

These results suggested that RV-D1 suppresses inflammation in HFLSs via the p-38, NF-κB, and AKT signaling pathways (Fig. 6).

## Discussion

OA represents the greatest joint pathology, and it is ubiquitous and pivotal in its contribution to disablement (Page *et al*. 2011). Advancements in the therapeutic setting of this musculoskeletal disorder are of great importance, with objectives of developing both prophylaxis and remediation strategies. Joint inflammation indicates the initiation of OA. In the early stages of articular inflammation, the innate immune system produces a response that elicits the recruitment and activation of leukocytes and neutrophils. Matrix fragments and by-products stimulate the innate immune cascade via receptors present in the synovium. Subsequent cascades are regulated by NF-κB, a principal protein, whereby the synovial milieu induces the synthesis of potent proinflammatory mediators, including MMP-3 and MMP-13, leading to degradation of cartilaginous structures. RV-D1 functions as an endogenous lipid mediator synthesized during the resolution phase of inflammation and is known for its robust pro-regenerative and anti-inflammatory attributes (Norling *et al*. 2016). The key finding from our experiments elucidates the pivotal role of RV-D1 in modulating critical mechanisms inherent in OA pathophysiology, encompassing the regulation of inflammatory mediators and MMPs within the synovial milieu. Our findings support the notion that ameliorating synovitis via RV-D1 administration can suppress cartilage degradation and lesion formation.

ALX/FPR2 represents a G protein-coupled receptor, characterized by its complex capacity to induce diverse cellular reactions. RV-D1 is recognized for its affinity to ALX/FPR2, eliciting anti-inflammatory and regenerative responses, and it triggers a spectrum of intracellular signaling cascades. Lipoxin, an active autacoid derivative of arachidonic acid, promotes FPR2 homodimerization, thereby facilitating pro-resolution signaling predominantly via suppression of NF-κB-associated proinflammatory pathways (Qin *et al*. 2022). Conversely, GPR32 also functions as a G protein-coupled receptor and is known for its presence on various immune cell types including monocytes, leukocytes (Krishnamoorthy *et al*. 2010),(Chiurchiù *et al*. 2016), and cutaneous immune cells. However, no expression of GPR32 was detected on HFLSs in our experimental setting.

IL-1β is considered a fundamental pathogenic mediator in OA. IL-1β overproduction induces the activation of synovial cells and chondrocytes, triggering the release of prostaglandin E2, MMPs, and pro-inflammatory cytokines, thereby inducing synovial inflammation and potentially disrupting the cartilaginous matrix. Conversely, IL-1β affects endothelial cells and induces the recruitment of neutrophils, lymphocytes, and macrophages, thereby enhancing the local inflammatory cascade in joints (Dong *et al*. 2020),(Nicolau *et al*. 2017). MMP-3 promotes collagen degradation and significantly contributes to cartilage deterioration (Wisithphrom *et al*. 2006). Therefore, it is imperative to develop strategies for suppressing MMP-3 hyperactivity to ameliorate synovitis and cartilage degradation. MMP-13, an enzyme that promotes the degradation of collagen types I, II, and III, is a key factor in OA pathogenesis. Notably, surgical intervention via the surgical destabilization of the medial meniscus in *Mmp13* knockout mice reduces the susceptibility to cartilage degeneration and erosion (Little *et al*. 2009). Given its pivotal role in OA pathogenesis, MMP-13 was chosen in this investigation. Consequently, we aimed to assess the impact of RV-D1, renowned for its anti-inflammatory properties, on IL-1β-induced production of MMP-3 and MMP-13. We found for the first time that the increased gene and protein expression of MMP-3 and MMP-13 in IL-1β-treated HFLSs was suppressed by treatment with RV-D1. This observation aligns with prior findings indicating that RV-D1 attenuates IL-1β-induced increases in MMP-3 production in periodontal ligament fibroblasts (Zarrough *et al*. 2023) and with additional studies on fibroblasts demonstrating that RV-D1 treatment reduces MMP-3 expression in a murine emphysema model (Posso *et al*. 2018). Given that MMP-3 stimulates MMP-13 and considering the central role of MMP-13 in the MMP activation cascade, it was a significant revelation that RV-D1 treatment inhibited the expression of both MMP-3 and MMP-13.

IL-1β induces the activation of NF-κB and MAPK signaling pathways, which participate in and regulate the expression of multiple genes involved in inflammation (Chauhan *et al*. 2021). The signaling pathway molecules can be activated by various post-translational modifications, such as phosphorylation, which consequently enhance innate immune responses. Thus, inhibiting the activation of these factors helps control inflammation in several diseases. The AKT pathway is known for its pivotal role in regulating various cellular functions, including metabolism, cell growth, proliferation, survival, transcription, and protein synthesis. In the Rheumatoid arthritis (RA) Fibroblast-like synoviocytes, AKT phosphorylation is essential for the secretion of MMP-3 upon IL-1β stimulation (Tian *et al*. 2013). In the present study, we evaluated AKT signaling in the anti-inflammatory effect of RV-D1. AKT phosphorylation activated by IL-1β stimulation was suppressed via RV-D1 treatment. The results suggest that RV-D1 may suppress the expression of MMP-3 and MMP-13 by regulating the AKT pathway. In our study, RV-D1 treatment decreased the phosphorylation of p-38, NF-κB, and AKT signaling pathway proteins in HFLSs, thereby reducing the production of IL-1β, MMP-3, and MMP-13. Furthermore, WRW4, an antagonist of ALX/ FPR2, treatment weakened the effect of RV-D1, resulting in p-38, NF-κB, and AKT phosphorylation and the protein expression of MMP-13 at levels comparable to those in the IL-1β without RV-D1 (Fig. 5). These results indicate that RV-D1 binds to ALX/ FPR2 and suppresses MMPs expression by regulating p-38, NF-κB and AKT signaling in inflamed HFLs. Although these results can partly explain how RV-D1 functions as a protective regulator in inflammatory HFLSs, the further investigation is needed to uncover the precise mechanisms by which these pathways regulate their downstream signaling.

In conclusion, RV-D1 suppressed IL-1β and MMP expression by inhibiting the phosphorylation of p-38, NF-κB, and AKT in inflammation in HFLSs. RV-D1 can be used to develop treatments for OA and other inflammatory disorders.

## Data Availability

Data could be obtained upon reasonable request to the corresponding author.
